# Tough surroundings make cancer tougher for immune cells to fight

**DOI:** 10.1038/s42003-023-05756-4

**Published:** 2024-01-09

**Authors:** Joel P. Joseph

**Affiliations:** Department of Bioengineering, Indian Institute of Science, Bengaluru, India

## Abstract

The physical properties of the cells and proteins surrounding a tumor play a crucial role in determining the spread of the cancer. How they change cancer cells to suppress the immune response against them is an interesting question addressed in a recent study from Liu et al., which describes how increased stiffness of the tissue around the tumor decreases the amount of a protein—cyclic GMP-AMP synthase—in cancer cells, ultimately blocking the immune response to cancer.

**Figure:**
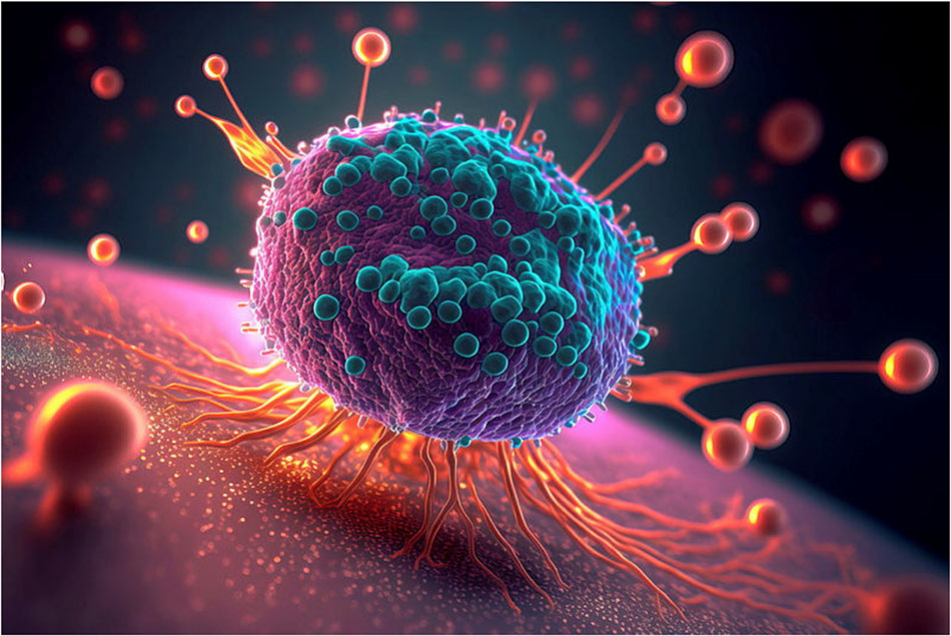
Лилия Захарчук, stock.adobe.com

The physical properties of the tumor microenvironment—cells and proteins surrounding the tumor cells—contribute to the worsening of cancer^1^. Immune cells typically recognize tumor cells based on their biochemical changes, e.g., changes in the structure or abundance of some proteins on their surface. This elicits an appropriate immune response to kill tumor cells. However, tumor cells and the tumor microenvironment can trick the immune cells^2^. Previous studies have identified the stiffening of the extracellular matrix (ECM), a meshwork of proteins in which the cells in our body’s tissues are embedded, to be one such factor^3^. In a recent study in *Cell Reports*, Liu et al. describe how this change in ECM stiffness in the tumor microenvironment translates to biochemical changes in tumor cells, which ultimately helps them evade the immune response^4^.

The authors cultured breast and liver cancer cells in soft and stiff matrices to understand the effects of ECM stiffness on cancer cells. Specifically, they tested the ability of the cancer cells cultured in these matrices to activate immune cells—dendritic cells and T cells. As hypothesized, the tumor cells cultured in the soft matrix could activate immune cells, but those cultured in the stiff matrix could not. To unravel the mechanism underlying this phenomenon, the authors investigated proteins and pathways that could translate the change in ECM stiffness in the tissue microenvironment to biochemical changes within the tumor cells. Consequently, they identified that ECM stiffening activated a protein, namely non-muscle myosin II A, which in turn assembled another protein called F-actin to form stress fibers within the cell. Rho-associated coiled-coil containing protein kinase (ROCK), an activator of myosin IIA, triggers the chain of events. On the other end, the stress fibers deprive the cells of another protein, TRIM14. Consequently, the cyclic GMP-AMP synthase (cGAS) protein gets degraded in the cell via the known autophagy-based protein degradation pathway. Low levels of cGAS, a protein that senses DNA in the cytoplasm of the cell, cause decreased production of a messenger molecule that triggers a cascade of reactions to activate immune cells — cGAMP. In this way, ECM stiffening reduces the ability of tumor cells to elicit an immune response, thus allowing for an unchecked spread of cancer. The researchers not only tested this hypothesis in cell lines but also validated them in mouse models of cancer, human breast and liver cancer tissues, and genomic analysis. They found proteins related to the pathways described above, e.g., myosin, to be abundant in these cancer models. Moreover, blocking the formation of ROCK-myosin mediated actin stress fibers reduced tumor size in mice and caused both cell lines and tumor-bearing mice to activate immune cells.

This study adds to the emerging appreciation of how the physical properties of the tumor microenvironment can play a role in advancing the tumor. It identifies a specific pathway by which a physical property changes cancer cells and reduces their ability to trigger immune cells to elicit a response, thus evading the immune system. This finding could enable researchers to identify molecules that could target the proteins in this pathway to make the cancer cells more susceptible to immune response, possibly alleviating the disease. However, finer details of other molecular players that may be involved in the process must be studied. Further, as the authors mention, researchers are yet to investigate whether the chemical agents used to block myosin IIA-F actin fiber formation could directly influence immune cells and contribute to the observations that they have made.
